# Feast–Famine in Cyclic Autotrophy/Heterotrophy Doubles Microalgal Productivity while Controlling Bacterial Contamination

**DOI:** 10.1021/acssuschemeng.5c05990

**Published:** 2025-09-26

**Authors:** Fabrizio Di Caprio, Flavia Del Signore, Laura Capobianco, Francesca Pagnanelli, Pietro Altimari

**Affiliations:** Dipartimento di Chimica, Università Sapienza di Roma, Piazzale Aldo Moro 5, 00185 Rome, Italy

**Keywords:** wastewater treatment, lutein, feast and famine, process control, cheese whey, *Chlorella sorokiniana*, kinetic models, circular economy

## Abstract

Conventional outdoor microalgal cultures are limited by light availability, with the absence of light during the night causing approximately 50% reduction of daily productivity in Europe. In this study, an innovative process was developed to enhance microalgal biomass productivity in photobioreactors by supplementing organic substrate at sunset, thereby implementing a cyclic autotrophic/heterotrophic cultivation (day, autotrophic; night, heterotrophic). When organic substrate was supplied at sunset in quantities sufficient to sustain growth throughout the whole night, significant bacterial contamination occurred, leading to a yield *Y*_X/S_ = 0.18 g g^–1^. To address this issue, a “feast–famine” feeding strategy was here designed, optimizing substrate dosage and supplementation frequency based on kinetic models predicting both microalgae and bacteria growth. Under 12 h/12 h day/night cycles, this optimized strategy nearly doubled biomass productivity, from 0.68 to 1.28 g L^–1^ day^–1^, while keeping bacterial contamination negligible (comparable to autotrophic control) improving *Y*_X/S_ to 0.54 g g^–1^. Using cheese whey as a source of organic substrate resulted in a modest increase in biomass productivity and lower yield. This study provides general guidelines for designing effective organic substrate feeding strategies to enhance microalgae biomass productivity under cyclic autotrophic/heterotrophic cultivation while keeping bacterial contamination below prescribed threshold levels.

## Graphical abstract

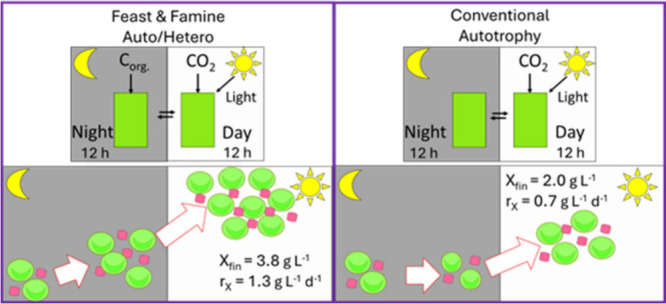

## Introduction

Microalgae are photosynthetic microorganisms that have emerged as promising systems for the production of food, feed, and other bio-based products. However, their widespread application in many economic sectors, such as biofuels, biomaterials, and feed, is currently limited by the high production costs of microalgae biomass, which can be primarily attributed to low biomass productivity in industrial plants.^1^ Microalgae can grow through various metabolic pathways: autotrophic metabolism, which uses light as the energy source and CO_2_ as the carbon source; chemoheterotrophic metabolism (hereafter referred to simply as heterotrophic), in which organic substrates serve as both carbon and energy sources; or mixotrophy, a combination of both metabolic processes. Autotrophic cultivation in outdoor photobioreactors is currently the most widely used industrial approach, but the limited availability of light remains a critical issue. Because of day–night cycles, outdoor industrial plants receive light for approximately half of the operating time in many geographical areas, as in Europe, which significantly reduces the biomass productivity compared to constant illumination.^2^ In particular, during night, when light is unavailable, microalgae cannot grow and biomass concentration can decrease by 1–25% due to maintenance metabolism,^3^ primarily driven by the consumption of storage compounds (starch and lipids) as energy source.^4^ This unproductive period results in a waste of energy used to keep the mixing pumps, aeration pumps, and temperature control system running.

Feeding photobioreactors with organic substrates at sunset can stimulate a heterotrophic light-independent metabolism during the night, compensating for the absence of light and enhancing biomass productivity and energy efficiency of the process.^5^ This configuration, named cyclic autotrophic/heterotrophic cultivation,^5^ may reduce energy requirements for photobioreactors by 40%, albeit with the drawback of higher land occupation requirement when the organic substrate is derived from conventional cultures (e.g., sugar beet).^6^ For this reason, this strategy appears particularly promising when wastewater or agro-industrial byproducts are used as a source of organic carbon. Although this strategy might seem easy to implement, the main bottleneck is the control of bacterial contamination.^7^ In fact, typical outdoor photobioreactors are not designed to operate under axenic conditions, and microalgae are typically cultivated alongside their microbial flora, mainly composed of symbiotic heterotrophic bacteria.^8^ In purely autotrophic cultures, this flora is generally kept under control due to the limited concentration of organic carbon in common photobioreactors, which mainly consist of extracellular products released by microalgal metabolism. However, if photobioreactors are supplemented with larger amounts of external organic substrate, bacteria can rapidly become the predominant biomass, as their specific growth rates are typically higher than those of microalgae.^9,10^ The idea of organic supplementation during the night has been tested in previous studies,^5,11^ yielding significant improvements in microalgae growth rate. However, little attention has been paid to the issue of bacterial contamination, which could hinder full-scale application. Previous studies that tested organic carbon supplementation at night used axenic cultures and concluded that the development of effective strategies to control bacteria contamination is the main challenge to overcome for scaling up.^5,11^ There is a scientific gap concerning the possibility of controlling bacterial contamination in xenic microalgae cultures fed with organic substrate during the night in day–night cycles. To date, the supply of organic substrate to photobioreactors has mostly been conducted using axenic cultures at the laboratory scale^7,11,12^ or xenic extremophile microalgae, such as *Galdieria sulphuraria*.^13^ However, for common microalgal species used and approved in the food sector, such as *Chlorella*, attempts to feed organic substrate to photobioreactor have often led to contamination issues.^7,9^ Developing strategies to control bacterial contamination in photobioreactor supplemented with organic substrates is currently a key unsolved challenge for the scale-up.^7^ The development of a strategy for this purpose is the main aim of this study. A previous study under fully heterotrophic conditions demonstrated that bacterial contamination can be effectively controlled in microalgae cultures using a “feast and famine” strategy, whereby after organic substrate supply (feast phase), a period of starvation follows (absence of organic substrate), which induces the decay of bacterial cell concentration (famine phase).^10^ In this study, these principles are applied to design an innovative cyclic autotrophic/heterotrophic cultivation strategy. Organic substrate is supplied during the night in calibrated amounts, calculated using growth models, to ensure depletion by sunrise. During the following diurnal hours, the reactor operates under organic substrate depletion, inducing a famine phase for bacteria, while microalgae can continue growing via photosynthetic metabolism. In this way, this strategy has the additional feature to be a true feast and famine process only for the heterotrophic bacteria, while microalgae have the additional advantage to grow during both night (using an organic substrate) and day (using light).

This study aims to demonstrate, for the first time, that bacterial contamination can be maintained below prescribed threshold limits while doubling microalgal biomass productivity in cyclic autotrophic/heterotrophic cultivation of xenic microalgal cultures under day/night cycles.

## Materials and Methods

### Microalgal Strain and Maintenance Conditions

*Chlorella sorokiniana* (*C. sorokiniana*) SAG 211/8k was maintained in 300 mL Erlenmeyer flasks, in xenic conditions, with 150 rpm orbital shaking, in BG-11 medium, with 24 h/24 h irradiation (1:3 blue:red LED lights, where blue = 400–520 nm and red = 610–720 nm; Roleandro HY-G-40W) at 80 μmol m^–2^ s^–1^ and at 25 ± 3 °C. From flasks, microalgae were inoculated with 500 mL of modified M-8 medium (Supporting Information Table S1) into two (*h* = 35 cm; *d* = 5 cm) column glass photobioreactors (PBRs) at 0.1 g L^–1^. Temperature was maintained at 25 ± 1 °C and pH at 6.7 ± 0.5 (by a CO_2_ supply). PBRs were fed with 1 L min^–1^ of air (filtered at 0.2 μm) and 35 mL min^–1^ of pure CO_2_, under a day–night cycle of 12 h/12 h. Light was provided by LED lamps (GROWSTAR L-QB1, sunlight full spectrum) supplying 500 ± 50 μmol m^–2^ s^–1^ photons (measured with a Gossen Mavolux digital luxmeter at three different heights of the reactors; conversion factor, 0.0257). *C. sorokiniana* was maintained in PBRs for sequential batches at concentration between 0.1 and 3.0 g L^–1^, to be maintained in exponential growth. Before starting experiments, at least two consecutive batches of 3 days were carried out in these conditions to adapt algae to the PBRs.

*C. sorokiniana* was chosen for this study as a representative species for commercial microalgae because *Chlorella* strains are already widely cultivated on an industrial scale. *C. sorokiniana* is approved for use as food in Europe, it is a fast-growing strain capable of tolerating high light and high temperatures, and it can use different substrates and metabolic pathways for growth.^12,14,15^

### Determination of Specific Growth Rates

Specific growth rate (μ, h^–1^) for microalgae was determined during their maintenance in PBRs, by using experimental growth data and by model fitting with eq 1.^16^

$$\ln X\left(t\right)=\ln X_{0}+\mu t$$ 1

with *X*(*t*) denoting the biomass concentration at time *t* and *X*_0_ the biomass concentration at the beginning of the cultivation, as g L^–1^. The fitting was carried out including either all data of the batch (i.e., including both the periods of light availability and darkness) or only data from daytime (i.e., only the period of light availability) to calculate μ_mean_ and μ_max_, respectively.

The specific growth rate of heterotrophic bacteria flora (μ_B_, h^–1^) living in xenic microalgae cultures was determined by a dedicated experiment in which two PBRs were inoculated with 0.1 g L^–1^ xenic microalgae inoculum and fed with 10 g L^–1^ pure glucose or galactose, at 25 ± 1 °C, under dark condition and 1 L min^–1^ air supply rate. Bacteria cell concentration (cell mL^–1^) was monitored at different sampling points, and eq 1 was used to determine μ_B_ using data in the exponential phase. The doubling time (*t*_d_, h) of cells was calculated by using eq 2:

$$t_{d}=\frac{\ln\left(2\right)}{\mu }$$ 2

To determine the heterotrophic growth rate of *C. sorokiniana* on glucose and galactose, dedicated experiments under axenic conditions were operated in 250 mL flasks, with 100 mL of culture medium, in dark condition, under 150 rpm orbital shaking, at 25 ± 1 °C, with 10 g L^–1^ glucose or galactose in the culture medium. For both microalgae and bacteria, cell concentrations were determined by flow cytometry.

### Cheese Whey Collection and Pretreatment

Cheese whey was collected after the production of ricotta cheese (precipitation of whey proteins at 85–90 °C) from Campo Felice cheese factory (AQ, Italy). Before processing, the milk was pretreated with lactase to split lactose into d-glucose and d-galactose. Cheese whey was ultrafiltered with the membrane model GM2540F (2 nm pore size) as described in a previous work.^10^ Cheese whey ultrafiltration permeate (*P*_UF_) was stored at −18 °C until its utilization as feed for microalgae cultivations. Cheese whey was chosen as waste in this study because it contains mainly sugars, as an organic substrate, available for microalgae metabolism. The chemical composition of *P*_UF_ is reported in the Supporting Information ( Table S3).

### Implementation of the Different Feeding Strategies

The main experiments carried out in this study involved the operation and comparison of three different feeding strategies of organic substrate to microalgae cultivated in PBRs. These strategies have been compared with a control in autotrophic conditions (no feed of organic substrate). The organic carbon substrates used in this study as fed were pure glucose and *P*_UF_.

Microalgae from maintenance PBRs were inoculated at 0.1 g L^–1^ into 500 mL of PBRs at the beginning of the experiment. In what follows, the different feeding strategies implemented to operate the reactors are described in detail:

(1) *Autotrophy (PA)*: Reactors were operated as already described for maintenance.

(2) *Cyclic autotrophy/heterotrophy with glucose (G)*: Microalgae were cultivated in the modified M8 medium with glucose supplied at the beginning of every night period (from a 500 g L^–1^ stock solution) in the quantity predicted to be consumed within sunrise, (i.e., within the duration of the night period Δ*t* = 12 h). To this purpose, the concentration of supplied glucose (Δ_glu_, g L^–1^) was calculated using eq 3:^17^

$$\Delta_{\mathrm{glu}}=\frac{X_{e,n}-X_{s}}{Y_{X/S}}$$ 3

where *Y*_X/S_ is the biomass to glucose yield factor, which was set at 0.50 g_X_ g_S_^–1^. Typical *Y*_X/S_ for *C. sorokiniana* is 0.4 g_X_ g_S_^–1^;^12,18^ here 0.5 g_X_ g_S_^–1^ is set to ensure that no residual glucose accumulated in the medium at the end of the night phase. *X*_s_ (g L^–1^) is the biomass concentration measured at sunset, namely, at the last sampling point before glucose feeding and light being switched off, and *X*_e,n_ (g L^–1^) is the predicted biomass concentration at the end of the night period, determined using eq 4:^17^

$$X_{e,n}=X_{s}e^{\mu \left(\Delta t\right)}$$ 4

This calculation was made assuming there was no significant bacteria growth during the night, using *Y*_X/S_ and μ estimated for microalgae, and Δ*t* = 12 h.

(3) *Cyclic autotrophy/heterotrophy with controlled glucose supply (GCC)*: Microalgae were cultivated in the modified M8 medium supplied with glucose. The approach to supply glucose was similar to that adopted in the implementation of the strategy G but with two relevant differences. The first difference was that glucose was supplied in lower amounts, to be depleted within 9 h (Δ*t* = 9 h). This different time was set based on simulations, to reduce bacteria contamination. The reasons for this choice are discussed in detail in Results and Discussion. The second difference was that, after glucose depletion, the second glucose supplementation was made after a time interval of 35 h. This prolonged time interval was set based on previous results reporting the kinetics of bacteria cell decay under energy starvation condition.^10^

(4) *Cyclic autotrophy/heterotrophy with wastewater supply (CWUP)*: Microalgae were cultivated in the modified M8 medium following the same strategy as that described for GCC. The only difference was that *P*_UF_ was used as the organic substrate source in place of pure glucose, with an amount of carbohydrates supplied equivalent to the strategy GCC.

Each different strategy was tested in biological replicates into separate PBRs (*n* = 4 for G and *n* = 2 for PA, GCC, and CWUP). All experiments were conducted for 3 days, corresponding to three consecutive day/night cycles.

### Determination of Biomass and Cell Concentration

Biomass concentration (microalgae + bacteria) was determined by filtering a known volume through 0.2 μm cellulose acetate filter, which was then dried at 105 °C. Microalgae and bacteria cell concentrations were measured through flow cytometry (Attune NxT flow cytometer, Thermo Fisher Scientific). Before analysis, the reproducibility of the instrument was verified by using Attune Performance tracking beads. Samples were diluted to (2.5–5) × 10^6^ cells mL^–1^ using TE buffer (10 mM Trizma base Sigma-Aldrich, 1 mM disodium EDTA, pH 8) and subsequently diluted with 1% glutaraldehyde at 4 °C for 1 h. Then samples were stained by adding 1 μL of SYBR-Green I 300x (in dimethyl sulfoxide) to 300 μL of fixed sample and incubating it in the dark for 15 min at 25 °C. Samples were then diluted again in TE buffer to 5000–20000 microalgal cells mL^–1^ and 70000–350000 bacterial cells mL^–1^. The samples were finally analyzed by flow cytometry acquiring a 50 μL sample at a flow rate of 100 μL min^–1^. Plotting the signal from chlorophyll red fluorescence emission detected by BL3 filter (BP 695/40), vs the green fluorescence signal of the SYBR-Green I detected by the BL1 filter (BP 530/30), bacteria population and microalgae population were separated. Further information about the protocol can be found in a previous work.^10^

Bacteria contamination was quantified using eq 5.

$$f_{B}=\frac{n_{B}}{n_{M}}$$ 5

where *n*_M_ and *n*_B_ are respectively the microalgal and bacterial cell concentrations as cells mL^–1^.

The mean cell mass of *C. sorokiniana* was calculated as the ratio between biomass concentration and cell concentration based on samples collected from autotrophic cultivation.

### Chemical and Biochemical Analysis

Glucose concentration was measured by a colorimetric method using the GOPOD reagent (glucose oxidase/peroxidase) from the Megazyme d-Glucose Assay Kit. Total sugar concentration was determined by the phenol–sulfuric acid (Dubois) method.^19^ Galactose concentration was estimated by the difference between the concentration of total sugars and glucose. Total nitrogen (TN) was analyzed by the official method for water analysis by IRSA-CNR 4060 as previously described.^20^ Lutein content in the biomass was determined by centrifuging the collected sample (5 min, 13000 rpm); then the pellet was suspended in methanol and heated at 70 °C for 10 min. The sample was centrifuged again, and the supernatant was collected. The extraction steps were repeated until biomass discoloration. The extract was filtered with a polytetrafluoroethylene (PTFE) membrane filter (0.20 μm) and analyzed with high-performance liquid chromatography (HPLC) (SpectraSYSTEM HPLC, Thermo Fisher Scientific) using the Thermo Scientific Acclaim C30 column (4.6 mm × 150 mm, 5 μm) and UV–vis detector set at 450 nm. Elution was carried out using acetonitrile, 1:1 methanol:ethyl acetate (v v^–1^), and 200 mM acetic acid, at 1.50 mL min^–1^, following the procedure reported in Table S2. Lutein content in samples was determined using a standard curve obtained with a lutein standard purchased from Sigma-Aldrich (cod. 07168).

### Calculation of Process Parameters

Biomass productivity (*r*_X_, g L^–1^ h^–1^) was determined with eq 6.

$$r_{X}\left(t\right)=\frac{X(t)-X_{0}}{t}$$ 6

where *X*_0_ and *X*(*t*) are the biomass concentrations (g L^–1^) at the beginning of the experiment and at the time (h). The final productivity determined with *t* corresponding to the end of the cultivation experiment is denoted as *r*_X,f_, while *r*_X,max_ denotes the maximum *r*_X_ attained in the experiment.

The observed biomass to glucose yield (*Y*_X/S_, g_X_ g_S_^–1^) was determined using eq 7:

$$Y_{X/S}=\frac{X_{f}-X_{f,\mathrm{photo}}}{S_{\mathrm{TOT}}}$$ 7

with *S*_TOT_ denoting the sum of the glucose supplemented to the reactor (g) divided by the final reactor volume (L), *X*_f_ the final biomass concentration (g L^–1^) in the test with glucose, and *X*_f,photo_ the final biomass concentration attained in the control test PA.

The specific growth rates for microalgae (μ_A,av_, h^–1^) and bacteria (μ_B,av_, h^–1^) were determined by linear regression of ln (cell_A_ cell_A,0_^–1^) and ln (cell_B_ cell_B,0_^–1^) data vs time, using all data collected for microalgae and bacteria cell concentrations throughout the batch. Lutein productivity (mg L^–1^ h^–1^) was calculated using eq 8:

$$r_{\mathrm{lutein}}=\frac{X_{f}\chi_{\mathrm{lut},f}-X_{0}\chi_{\mathrm{lut},0}}{t_{f}}$$ 8

with χ_lut,f_ and χ_lut,0_ being the lutein content (mg g^–1^) inside final biomass and the initial biomass, respectively, and *t*_f_ the duration of the experiment (h).

### Statistical Analysis

All tests were replicated in two or four biological replicates (cultivation in independent reactors). The results were reported as the mean ± standard deviation (SD). Significant differences (α = 0.05) among treatments were evaluated by using Student’s *t* test, one-way analysis of variance (ANOVA), and Tukey’s post hoc test, by using R and Microsoft Office Excel software.

## Results and Discussion

### Autotrophic Cultivation under Day–Night Cycles

A main limit of conventional autotrophic cultivation of microalgae is the growth rate limitation due to the light supply. This is particularly evident during night phases, in which the dark conditions induce energy starvation. At night, under such conditions, the growth rate drops to zero or to negative values, remarkably reducing the average growth rate. This is a well-known phenomenon described in previous studies.^4,5^ The reduction in the growth rate depends on the duration of the night phase. To quantify the impact of the night phase on *C. sorokiniana* cultures, a first autotrophic experiment was conducted including two consecutive 3-days batches with day/night cycles of 12 h/12 h (Figure 1). This fraction of the light period (50% of daytime) was set because it is representative of a typical plant operating in Southern Europe. The mean growth rate (μ_mean_) measured for the whole experiment was 0.042 ± 0.003 h^–1^, corresponding to *t*_d_ = 16 ± 1 h. This value falls in the 0.025–0.043 h^–1^ range, previously reported for *C. sorokiniana* in autotrophic conditions under day/night cycles at the same temperature.^21,22^ When the growth rate was calculated considering only the daytime, its value was remarkably higher: 0.13 ± 0.01 h^–1^ (*t*_d_ = 5.3 ± 0.4 d^–1^). This improvement in the growth rate is ∼3-fold. It should be noticed that only a 2-fold improvement would be expected, due to the 50% of time in the night phase. Here, the extra increment in the growth rate can be explained by considering that during the night phase the biomass concentration decreases, likely due to the consumption of stored reserve compounds (e.g., starch and lipids) as a source of energy.^3^ Under day–night cycles, in autotrophic regime, microalgae regulate their metabolism using carbon fixed by photosynthesis to accumulate starch, which increases from almost 0% at half-day to ∼20% dry weight (d.w.) at sunset.^4^ This starch is then used as a major source of energy during night,^4^ resulting in a loss of mass. This loss of biomass might be reduced in industrial plants by the temperature drop occurring during the night.^3^

**Figure 1:**
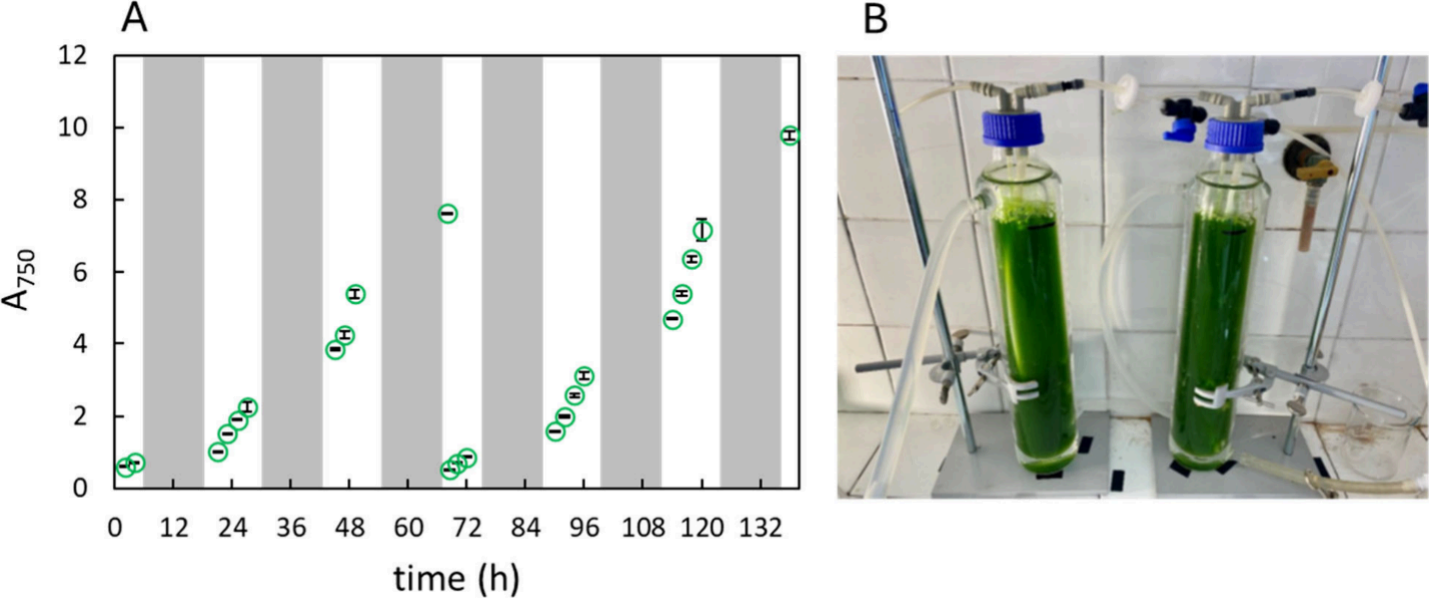
(A) Microalgae growth in autotrophic conditions for two sequential batches. Values are reported as the mean ± SD (*n* = 2). Day–night cycle was 12 h/12 h. Night phases are indicated in gray. (B) Photo-bioreactors used for cultivation.

### Supplementation of Organic Substrate at Night to Enhance Microalgae Growth Rate

The main idea investigated in this work is to develop a feeding strategy for supplementing organic substrates during the night to enhance microalgae biomass productivity in photobioreactors operated under day–night cycles. This idea is particularly attractive for industrial applications because it could allow overcoming the reduction in growth rate occurring at night, without changes in the design of the plant. Some previous studies already proved the possibility to enhance biomass productivity by supplementation of organic substrate at night, but the experiments were conducted with axenic cultures.^5^ The rise of bacterial contamination is the main issue that hinders the possibility of applying such strategies to real industrial plants, which are designed to work in xenic conditions.

The objective of this study is, therefore, to identify a feeding strategy that can also control bacterial contamination. The first strategy tested is similar to what reported in previous studies with axenic microalgae,^5,11^ and it is named strategy G: at the beginning of every night phase (sunset), an amount of glucose expected to be consumed within the following 12 h (i.e., the total night period) was supplemented. The amount of supplied glucose was predicted at each sunset based on the measured biomass concentration. Glucose is a substrate readily usable by *C. sorokiniana.* ^23^ It possesses three different active hexose transporters for its uptake.^24^ After glucose uptake, it can be quickly converted into energy and intermediate for the synthesis of new cells by glycolysis and oxidative pentose phosphate (OPP) pathway.^25^ Therefore, for the calculation, exponential growth was assumed to take place during the night (eq 4), with specific growth rate μ = 0.068 h^–1^, which was estimated based on *C. sorokiniana* cultivation under axenic conditions (Table 1). This calculation was made assuming that the bacterial contamination was negligible during the night phase. This approach of strategy G is comparable to the one followed in a previous study using a continuous reactor operated under axenic conditions.^11^ The results of testing strategy G are reported in Figure 3 and Table 2. The addition of the predicted amounts of glucose at sunset under 12 h/12 h day–night phases allowed increasing *X*_f_, *r*_X_, and *r*_X,max_ by a factor of 1.8 compared to the PA cultivation. However, the difference was not statistically significant (*p*-value between 0.052 and 0.06). This increment in biomass concentration is comparable to the increment obtained in a previous work in axenic conditions, in which glucose fed at night was calculated based on a fixed uptake rate (g_S_ g_X_ h^–1^)^5^ and higher than that achieved with continuous reactor.^11^ Glucose was completely depleted after each night phase (Figure 4). In total, 9 g L^–1^ glucose was added to the culture medium. Previous studies testing cyclic autotrophy/heterotrophy cultures to compensate for no biomass growth during the night were always conducted in sterilized reactors.^5,11^ Results in Figure 3B show that in an open reactor (xenic conditions) the increment in biomass concentration with strategy G (glucose available for the whole night) was obtained at the cost of relevant bacteria contamination. This contamination, quantified by the bacteria to microalgae cell concentration ratio, *f*_b_, attained a maximum *f*_b_ = 15 at 45 h and then decreased progressively. This maximum contamination was much higher (about 10-fold) compared to the PA control (*p* = 0.026), for which *f*_b_ was always ≤2.1. The high contamination was also confirmed by computing the average bacteria growth rate, which was significantly higher (lower *t*_d_) for the test G (*p* = 0.01) compared to the PA control (Table 2, Figure 5).

**Table 1:** Growth Rates of Bacteria Flora Living in *C. sorokiniana* Cultures and of Axenic *C. sorokiniana*, When Cultivated Heterotrophically on Glucose and on Galactose^a^

	Bacteria flora		*C. sorokiniana*	
	Galactose	Glucose	Galactose	Glucose
μ_max_ (h^–1^)	0.21 ± 0.01^a^ (*n* = 10)	0.22 ± 0.01^a^ (*n* = 14)	0.027 ± 0.002^b^ (*n* = 15)	0.068 ± 0.002^c^ (*n* = 13)

a Values are reported as mean ± standard error. Letters indicate statistically significant difference (*p* < 0.05).

**Table 2:** Final Biomass Concentration (*X*_fin_), Biomass Productivity Calculated for the Whole Batch (*r*_X,fin_), Maximum Biomass Productivity (*r*_X,max_), Biomass to Substrate Yield (*Y*_X/S_), Average Specific Growth Rate for Microalgae (μ_A,av_), and Bacteria (μ_b,av_) Calculated from All Data, Lutein Content in the Biomass Final Biomass (Lutein), and Final Lutein Productivity (*r*_lutein_)­^a^

	*X*_fin_ (g L^–1^)	*r*_X,fin_ (g L^–1^ d^–1^)	*r*_X,max_ (g L^–1^ d^–1^)	*Y*_X/S_ (g_X_ g_S_^–1^)	μ_A,av_ (day^–1^)	μ_B,av_ (day^–1^)	Lutein (mg g^–1^)	*r*_lutein_ (mg L^–1^ d^–1^)
PA	2.05 ± 0.05^a^	0.68 ± 0.02^a^	0.71 ± 0.02^a^		0.98 ± 0.07^a^	1.27 ± 0.03^a^	3.0 ± 0.3^a^	2.0 ± 0.2^a^
G	3.7 ± 0.8^ab^	1.3 ± 0.3^ab^	1.3 ± 0.3^ab^	0.18 ± 0.09^a^	1.23 ± 0.05^b^	2.0 ± 0.1^b^	4 ± 2^a^	5 ± 3^a^
GCC	3.78 ± 0.08^b^	1.28 ± 0.03^b^	1.28 ± 0.03^b^	0.54 ± 0.04^b^	1.24 ± 0.03^b^	1.48 ± 0.07^a^	2.5 ± 0.9^a^	3 ± 1^a^
CWUP	2.4 ± 0.2^ab^	0.79 ± 0.07^ab^	0.91 ± 0.00^ab^	0.25 ± 0.17^a^	0.81 ± 0.03^a^	1.6 ± 0.2^ab^	3.2 ± 0.5^a^	2.4 ± 0.3^a^

a Values are reported as mean ± SD (*n* = 2). Letters indicate statistically significant difference (*p* < 0.05).

It is worth observing that bacteria contamination resulted in a significant increment (*p* = 0.009) of the regression coefficient obtained by correlation of the optical density at 750 nm with the biomass concentration (see the Supporting Information, Figure S3). This method is extensively used for the estimation of the biomass concentration in bioreactors.^26^ However, these data show that using the regression coefficient determined for pure autotrophic cultures to estimate the biomass concentration in cultures contaminated by bacteria gives 32% underestimation of the biomass concentration. This result should be considered to prevent potential errors generated by using OD_750_ for estimating the biomass concentration in microalgae cultures when bacterial contamination is not adequately controlled.

Bacterial contamination became negligible for strategy G at the end of the cultivation, achieving a value (*f*_b_ = 2.2) comparable to that found for the PA control (*p* = 0.53). It should be emphasized that this reduction in the bacterial contamination was mainly observed during the third night phase, i.e., after the supply of glucose, and it cannot therefore be imputed to glucose depletion. The reduction in the bacterial concentration was rather determined by the appearance of a predator. In fact, an increment in the predator concentration, accompanied by a significant reduction in bacterial concentration, was observed with optical microscope for all four biological replicates with strategy G at the end of the cultivation experiment. Microalgae cell concentration did not show any decrement, in contrast, indicating that bacteria were selectively predated. Through microscopic observation, the predator was morphologically identified as a Ciliate, *Colpoda* sp.^27,28^ *Colpoda* sp. is ubiquitarian in the environment^27^ and commonly found in xenic *Chlorella* cultures.^29^ *Colpoda* species are bacterivores, as their diet mainly consist of bacteria. In a previous study, *Colpoda* was used to control bacteria proliferation in microalgae cultures, demonstrating its ability to selectively predate bacteria.^28^ This finding is of high practical interest, as it indicates the potential for using bacteria-specific predators to selectively remove bacterial contaminants from microalgal cultures.

On the other hand, even though the bacteria were predated during the second part of the cultivation experiment, they experienced a transient growth, consuming a relevant fraction of the supplied glucose, which could not be used by microalgae. As bacteria decayed due to predation, the resulting glucose-to-biomass yield factor *Y*_X/S_ = 0.18 (Table 2), which is 2- to 3-fold lower than typically values (0.38–0.50) measured for pure *C. sorokiniana*.^30,31^

Although glucose was completely consumed during the cultivation (−96%), an elevated final COD concentration (2500 mg L^–1^) was found in the culture medium. This COD, which cannot be imputed glucose, likely resulted from the degradation of bacteria biomass by the predator activity.

Lutein content inside biomass and lutein production rate did not vary significantly compared to the PA control. A previous study report a 34% decrease in lutein content with cyclic autotrophic/heterotrophic cultivation in continuous reactor, but the study did not provide experimental errors on lutein analysis.^11^

In summary, the glucose supplying strategy implemented during the test G allowed one to effectively increase biomass production as compared to the PA control, in agreement with what was reported in previous studies,^5,11^ but the xenic culture used in this study led to a relevant bacteria contamination, which reduced the substrate yield and may result in a biomass unusable for certain applications.

### Optimization of Glucose Supplementation to Control Bacterial Contamination by a Feast and Famine Strategy

The bacterial contamination observed with the G strategy was induced by the supply of glucose at sunset. Bacteria and microalgae competed for glucose during the night phase. After the glucose supply, it can be assumed that both bacteria and microalgae experience exponential growth until glucose is depleted (i.e., glucose is the stoichiometrically limiting nutrient). During this phase, since bacteria typically have higher growth rates than microalgae, *f*_b_ increases exponentially. Biomass concentrations of microalgae (*X*_M_) and bacteria (*X*_B_) over time can be predicted as follows:

$$X_{B}=X_{B,0}e^{\mu_{B}t}$$ 9

$$X_{M}=X_{M,0}e^{\mu_{M}t}$$ 10

from which the following expression can be derived for *f*_b_:^10^

$$f_{b,X}=\frac{X_{B}}{X_{M}}=\frac{X_{B,0}}{X_{M,0}}e^{\left(\mu_{B}-\mu_{M}\right)t}$$ 11

The glucose consumed can be computed by multiplying the produced biomass by *Y*_X/S_.

To implement the illustrated model, the following parameters are required: the initial concentrations at sunset (*X*_B,0_ and *X*_M,0_) and the growth rates (μ_M_ and μ_B_) of microalgae and bacteria. The μ_M_ values of axenic *C. sorokiniana* and μ_B_ values of bacteria flora living with the same algae were determined with independent experiments (Table 1). As expected, μ_B_ > μ_A_ (3-fold), in agreement with what was reported in previous studies.^10^ μ_A_ = 0.068 h^–1^ found for *C. sorokiniana* is comparable to the value previously reported for heterotrophic growth on glucose at 25–30 °C.^32^ *X*_B,0_/*X*_M,0_ can be estimated from *n*_B_/*n*_M_ measured under autotrophic conditions (Figure 3B) and used to calculate *n*_B,0_ for a known *n*_M,0_. During autotrophic cultivation, the bacterial flora consistently maintained a cell concentration comparable to that of *C. sorokiniana*, with a mean *f*_b_ of 1.3 ± 0.5. Therefore, to predict the amount of glucose to be supplied, the cell concentrations of microalgae and bacteria at sunset were assumed to always be equal (*f*_b_ = 1). This assumption was made based on the idea that the strategy targets the bacterial contamination control and aims to provide a strategy that does not require measuring bacterial concentration during cultivation. To convert cell concentration to biomass concentration, mean cell masses *m*_B_ = 1 pg cell^–1^ ^33^ and *m*_M_ = 20 pg cell^–1^ were considered for bacteria and *C. sorokiniana*, respectively. These data result in an initial bacterial contamination (*f*_b,X_) equal to 5% at sunset (start of the heterotrophic phase).

With these data, the temporal evolution of bacterial and microalgal biomass during the heterotrophic phase was simulated (Figure 2). After 12 h of heterotrophic exponential growth, the model predicts that bacteria become 20% of the total biomass, increasing up to 80% after 30 h. On the other hand, the duration of this phase is dictated by the amount of glucose supplied at sunset: decreasing the amount results in a shorter heterotrophic phase and, thus, in a reduced contamination. Accordingly, the amount of glucose can be computed to ensure that bacterial contamination does not exceed a prescribed threshold. The following procedure was implemented to predict the amount of glucose to be supplied to ensure the achievement of a maximum threshold *f*_b,X_:

(1) The time *t* required to achieve the prescribed contamination threshold was computed from eq 11 by setting *f*_b,X_ = 0.15.
(2) *X*_M,0_ and *X*_B,0_ were determined by multiplying *X*_0_ (biomass at sunset) by 0.95 and 0.05, respectively.
(3) The bacterial and the microalgal biomass concentrations as a function of time *t*, *X*_M_(*t*) and *X*_B_(*t*), were computed by eqs 9 and 10.
(4) The mass of glucose to be supplied was predicted by summing the masses of glucose needed to achieve *X*_M_(*t*) and *X*_B_(*t*). To this purpose, the same *Y*_X/S_ = 0.5 g g^–1^ was assumed for both microalgae and bacteria, yielding a mass of glucose equal to (*X*_M_(*t*)– *X*_M,0_(*t*) + *X*_B_(*t*)–*X*_B,0_(*t*))/*Y*_X/S_.

**Figure 2:**
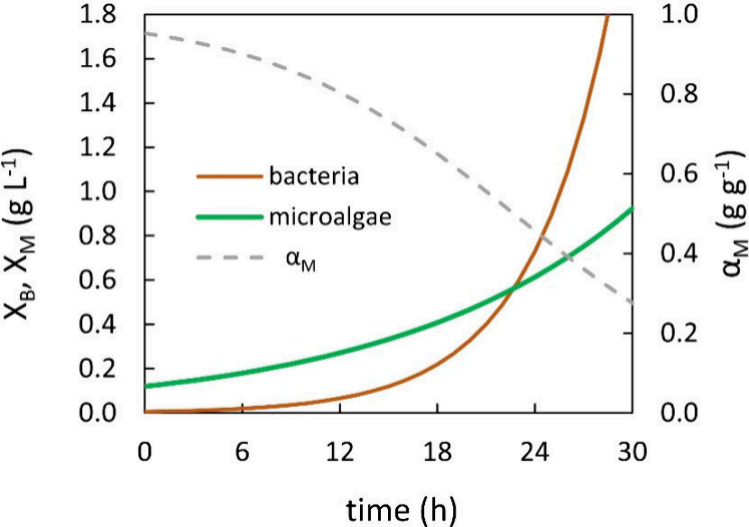
Prediction of bacteria (*X*_B_) and *C. sorokiniana* (*X*_A_) competition in terms of biomass evolution in a batch reactor supplemented with glucose in heterotrophic condition. Term $\alpha_{M}=\frac{X_{M}}{X_{M}+X_{B}}$ .

Through the application of the illustrated procedure, f_b,X_ = 0.15 was attained for *t* = 9 h, which is shorter than the 12 h used for the test G. In addition, also the famine phase (the phase without glucose) was modified in the test GCC. The results of a previous study indicated that when the organic substrate is depleted, bacteria decay dropping to about one-third in 24 h.^10^ Therefore, glucose was not supplied at every sunset to have a time window of at least 24 h without glucose. To this aim, only two additions of glucose (instead of 3 as for strategy G), were operated, with a time window without glucose set at 35 h between the first and the second heterotrophic phase (i.e., glucose was not added the night of the second day) (Figure 4B). These conditions have been implemented in the GCC strategy, whose results are reported in Figures 3 and 4 and Table 2. The GCC strategy allowed us to attain a biomass production and productivity comparable to those achieved in test G and significantly higher than the PA test (*p* = 0.048 for *X*_fin_ and 0.047 for *r*_X_), but with only 36% of the glucose supplied in test G (3.2 g L^–1^ in total). This is a remarkable increment in the efficiency of glucose utilization, which resulted in an overall *Y*_X/S_ = 0.54 ± 0.04 g_X_ g_S_^–1^, which is 3-fold higher than the 0.18 value obtained for strategy G (*p* = 0.02). This yield agrees with values previously reported for axenic heterotrophic *C. sorokiniana*, which are between 0.38 and 0.50 g_X_ g_S_^–1^.^30,31^ The higher *Y*_X/S_ was a result of improved contamination control with the strategy GCC. In fact, throughout the whole GCC cultivation, bacterial contamination was maintained at negligible levels, comparable to values attained by the PA control (Figure 3B,D), with *f*_b_ values significantly lower than those with the G strategy (*p* = 0.02). It should be noted that predators have been observed even in the GCC strategy. There was no evident decay of bacteria during the famine (day) phases. This latter result could be due to two reasons: (i) there was a time window of about 10 h after glucose depletion during which samples could not be collected due to technical limitations, thus missing the initial famine phase when a significant decline should have been observed; (ii) glucose starvation occurred mainly during the day, a phase when microalgae grew by photosynthesis. During this phase there is also a release of organic compounds by microalgae that can sustain bacteria growth at concentrations proportional to microalgae cells. Glucose was completely depleted at the end of the GCC test (−99.7%), and also the residual COD was negligible, at a value comparable to that of the PA control (Figure 4), indicating that glucose was mainly used for the production of microalgal biomass. These results demonstrate that a well-designed controlled supply of the organic substrate is fundamental to selectively producing microalgae biomass, increasing the productivity with respect to a phototrophic culture. It is important to underline how such results were achieved without uncoupling organic substrate and nitrogen, as made in previous studies.^20,34^ Indeed, nitrogen was always maintained at nonlimiting concentrations throughout the cultivation (Figure 4). High nitrogen in the medium allows the production of biomass with high protein content. The good control of bacteria growth was confirmed by the comparison of the average bacteria growth rates, which were not statistically different comparing GCC with PA control (*p* = 0.39), while significantly lower for GCC compared to G (*p* = 0.03). The heterotrophic phase during the night might also have improved photosynthetic efficiency during the day. This aspect should be investigated in dedicated studies. Lutein content inside the biomass and lutein productivity did not vary significantly as compared to the other cultivation tests. The content of lutein achieved at the end of the cultivation was in the range of 2.2–4 mg g^–1^ previously reported for fully heterotrophic cultivation of *C. sorokiniana,* ^35^ and higher than the 1.1 mg g^–1^ previously reported for cyclic autotrophic/heterotrophic cultivation with acetate.^11^ Lutein was analyzed because it is typically synthesized only by photosynthetic organisms^36^ and can therefore be considered a tracer of microalgae growth. Since its content in the biomass did not change significantly, it can be regarded as indicative of the production of high-quality microalgal biomass. A mass balance considering both the biomass produced and nitrogen consumed from the culture medium was used to estimate the content of nitrogen in the biomass. The nitrogen content was 9 ± 1%, with no significant difference among the different treatments (*p* = 0.72). This value is consistent with typical values reported for *C. sorokiniana,* ^37^ and corresponds to 43% protein content. A previous study showed a relevant decrease in protein content using a cyclic autotrophic/heterotrophic cultivation,^5^ but this was due to nitrogen depletion, which occurred earlier under conditions where higher biomass concentrations were attained. In this study, nitrogen was always present, and therefore no nitrogen depletion occurred. Other biochemical components were not analyzed; however, they are expected to remain substantially constant since all nutrients were available, and the estimated specific growth rates suggest no significant inhibition of microalgal cells.

**Figure 3:**
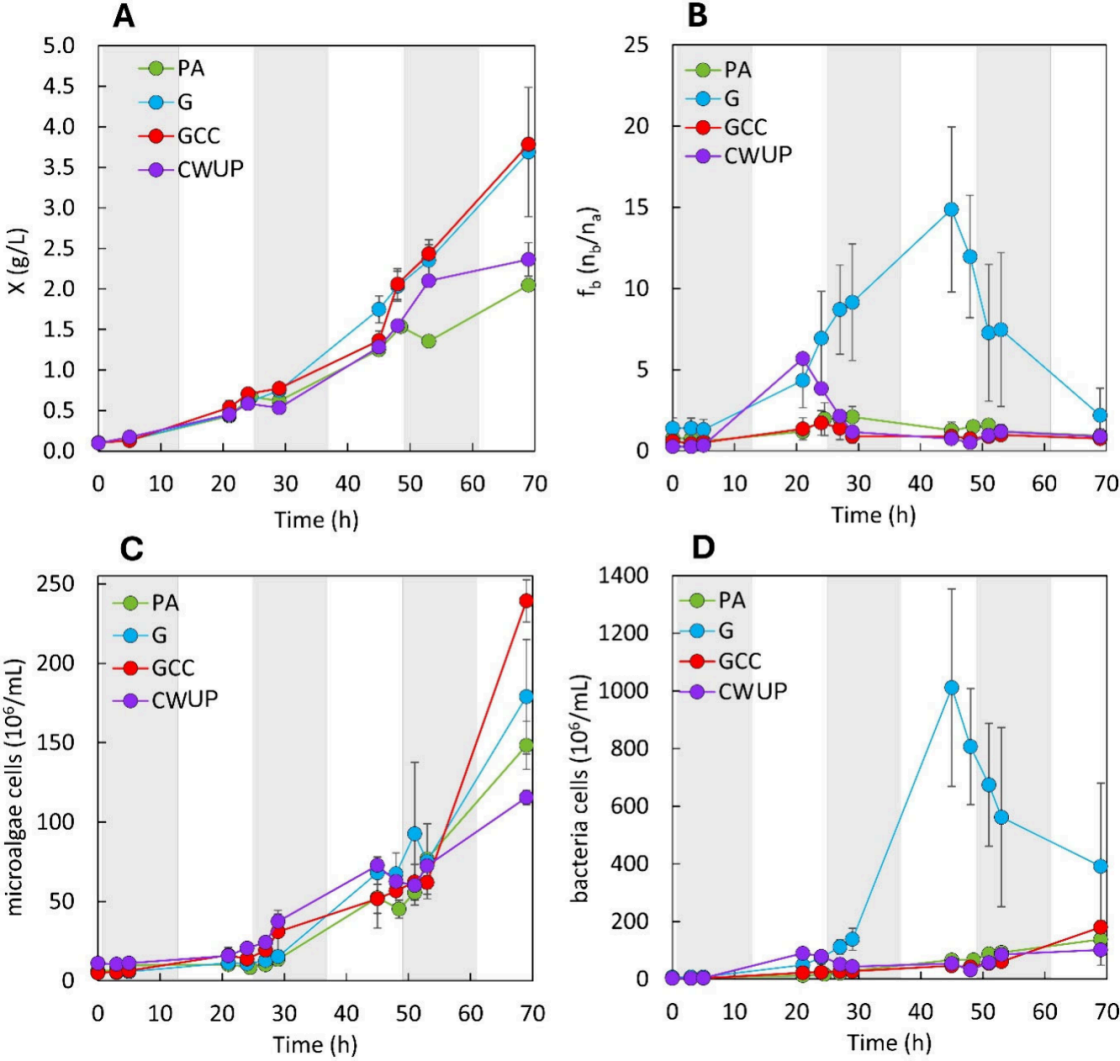
Total biomass (A), bacteria contamination (B), microalgae cells (C), and bacteria cells (D) in autotrophy (PA), cyclic autotrophy/heterotrophy with glucose supplemented at every sunset (G), cyclic autotrophy/heterotrophy with controlled glucose supply (GCC), and cyclic autotrophy/heterotrophy with controlled supply of cheese whey ultrafiltration permeate (CWUP). Values are reported as mean ± SD (*n* = 2). Day–night cycle = 12 h/12 h. Night phases are indicated in gray.

**Figure 4:**
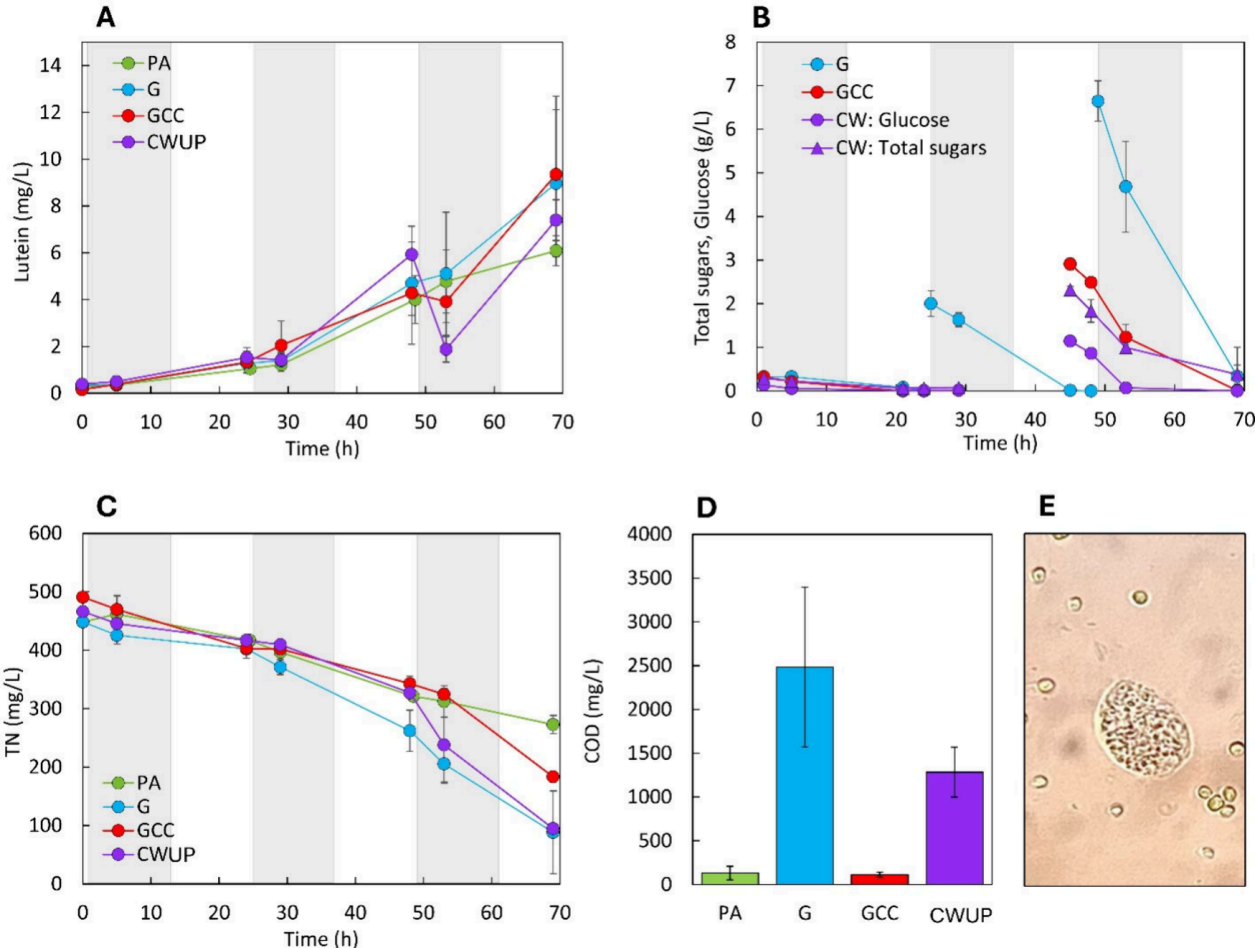
Lutein production (A), organic substrate (B), and total nitrogen (C) in the culture medium in autotrophy (PA), cyclic autotrophy/heterotrophy with glucose supplemented at every sunset (G), cyclic autotrophy/heterotrophy with controlled glucose supply (GCC), and cyclic autotrophy/heterotrophy with controlled supply of cheese whey ultrafiltration permeate (CWUP). (D) COD concentration at the end of the cultivation. (E) Predator found in the final sampling times of the test G. Values are reported as mean ± SD (*n* = 2). Day–night cycle = 12 h/12 h. Night phases are indicated in gray.

### Assessment of the Organic Substrate Supplementation Strategy Using a Real Wastewater

To verify the possibility to apply the same strategy to real wastewater, a final CWUP test was carried out using cheese whey ultrafiltration permeate as a source of organic substrate in place of pure glucose. The cyclic autotrophic/heterotrophic cultivation strategy had never been tested on real wastewaters before. The main obstacles related to the application of a real wastewater are the possible presence of further biological contaminants and the complexity of the organic carbon source, typically represented by different molecules, which might not be all usable by microalgae in their metabolism, or can induce inhibitory effects.^38^

Lactose, the main organic molecule in cheese whey, typically cannot be used by *Chlorella* species.^23,39^ *C. sorokiniana* is typically only able to use monosaccharides and unable to grow on lactose as a carbon source.^23^ For this reason, a specific stream was employed, which came from a production line in which milk was pretreated with lactase to split it into glucose and galactose. The whey employed was ultrafiltered to obtain *P*_UF_ and remove whey proteins, as they have a high nutritional and economical value and could not be used by microalgae as a carbon source. *P*_UF_ was supplied to microalgae cultures by following the same strategy as that described for the GCC test. The sum of glucose and galactose (total carbohydrates) was considered to calculate the amount of substrate provided. Potential further contamination by bacteria present in the *P*_UF_ was not considered, as the *P*_UF_ was stored in a freezer, and additional contaminants were assumed to be negligible. In terms of biomass production, the test CWUP allowed a +54% improvement in biomass concentration (*p* = 0.001) to be obtained after 54 h of cultivation (Figure 3A), while for all the other cultivation times sampled, biomass concentration was always comparable to that found during the PA control (*p* > 0.05). A +15% increase in biomass was obtained at the end of the test, compared to the autotrophic test, but the difference was not significant. With CWUP, bacterial contamination increased during the initial 20 h to values higher than the PA control (*p* = 0.002) and comparable to G. It then decreased over the following 10 h, attaining the same value as the control, which was maintained until the end of the batch. Such an initial excessive contamination was likely due to the presence of galactose, which is a poorer substrate than glucose for *C. sorokiniana.* In fact, *C. sorokiniana* has a μ_max_ = 0.027 h^–1^ on galactose, about 2.5-fold less than on glucose, while bacterial flora showed μ_max_ = 0.21 h^–1^ on galactose, which was comparable to μ_max_ on glucose (*p* = 0.48). The difference μ_B_ – μ_A_ for galactose is 0.18 h^–1^, higher than that for glucose (0.14 h^–1^). This result agrees with a previous work in which less efficient growth were reported for *C. sorokiniana* on galactose compared to glucose.^23^ Therefore, galactose gives a higher competitive advantage to bacteria than glucose. This heterogeneous response to substrates is a problem typically encountered when wastewaters are supplied to microalgae, as the ability of microalgae to use organic compounds is typically limited to a few molecules.^15,23,40^ Some substrates can have inhibitory effects on cells or can only be consumed when more readily available substrates are depleted (diauxic growth).^15^ In this context, from the perspective of pollutant removal, the presence of certain amounts of bacteria represents a positive factor, as it helps enhance removal efficiency. Glucose was completely depleted after *P*_UF_ addition (−99%), while the total carbohydrates supplied (2.6 g L^–1^) were depleted by −90% at the end of the test. Such removal could likely be increased with longer cultivation times. Indeed, when *C. sorokiniana* cells are in a medium containing different organic substrates, they typically exhibit diauxic growth, where they first use the most bioavailable substrate and then the other substrates.^41^ Therefore, microalgae first used glucose and then galactose, which require longer times for complete consumption. Final COD attained with the strategy CWUP was 1300 ± 300 mg L^–1^, about 3-fold higher than total carbohydrates, corresponding to 54% COD removal. This result suggests a partial conversion of sugars into organic fermentative products (e.g., lactic acid), in agreement with previous results.^10^ This phenomenon is detrimental for COD removal, but it might be beneficial if exploited for the production of other chemicals, as polyhydroxyalkanoates (PHA).^42,43^ Such conversion of sugars into fermentative products was likely responsible for the reduced *Y*_X/S_ observed for CWUP compared to that for GCC (Table 2). Since the substrate was converted to other organic molecules, it was only partially used for the production of biomass, and consequently, the measured *Y*_X/S_ = 0.27 was lower than the test GCC. The pH did not vary significantly among the different experiments; it slightly increased from the initial value of 6.7 to an average final value of 7.2, with no significant difference between the treatments. The average bacteria growth rate and *t*_d_ were not statistically different from those found in the other cultivation tests (Table 2, Figure 5). Lutein content and lutein productivity did not vary significantly as compared to the other cultivation tests.

**Figure 5:**
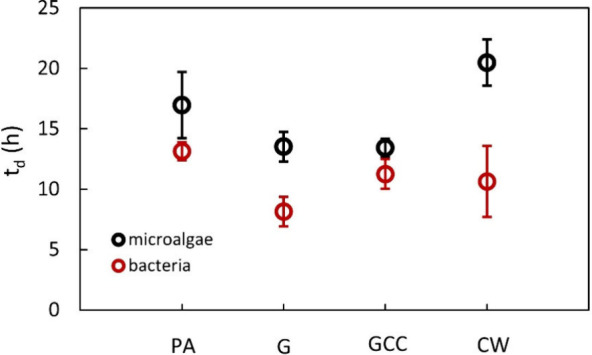
Average duplication time (*t*_d_) of bacteria flora and *C. sorokiniana* measured in the tested cultivation strategies: autotrophy (PA), cyclic autotrophy/heterotrophy with glucose supplemented at every sunset (G), cyclic autotrophy/heterotrophy with controlled glucose supply (GCC), and cyclic autotrophy/heterotrophy with controlled supply of cheese whey ultrafiltration permeate (CWUP). Values are reported as mean ± SD (*n* = 2).

Compared to the use of pure glucose (GCC strategy), feeding *P*_UF_ as an organic substrate source induces less significant improvements in biomass concentrations, mainly due to the lower yield and rate of microalgae using galactose as a substrate. These reduced improvements could be partially offset by the lower cost of the organic substrate and the pollutant removal service. However, to apply this strategy to real wastewater or byproducts, it is recommended to use wastewater containing an organic substrate that can be utilized with better efficiency (e.g., only glucose, or acetate).^12^

### Guidelines to Replicate the Cyclic Autotrophic/Heterotrophic Cultivation Process

The obtained results are promising for industrial scale-up, due to the doubling of the biomass production rate that was achieved with *C. sorokiniana* in conventional photobioreactors. The strategy GCC was the most effective one, which could be applied to conventional phototrophic plants. To apply and replicate this strategy to a certain cultivation, the following steps should be followed:

(1) experimental determination of the μ_A_ and μ_B_ and of the *Y*_X/S_ for the specific substrate to be used and for the specific microalga and bacterial flora, under growth conditions (temperature, pH, mineral medium) as close as possible to those of the cyclic autotrophy/heterotrophy;
(2) determination of the duration of the heterotrophic phase during the night by using eq 11, setting initial contamination (*X*_B,0_/*X*_A,0_) equal to the values measured in control autotrophic cultures and the final threshold contamination (*X*_B_/*X*_A_) to be achieved at the end of the heterotrophic phase (it should be noted that it could be even considered the possibility to implement a monitoring of the bacteria concentration in the reactors attained at sunset in order to have more accurate data for *X*_M,0_ and *X*_B,0_);
(3) determination of the amount of organic substrate to be supplied at sunset, at alternate days (at least 24 h between two supplies) from the value of biomass concentration determined at sunset.

## Conclusions

The application of the organic substrate supplementation strategy developed in this study significantly enhances the biomass productivity of conventional photobioreactors, approximately doubling it (from 0.68 to 1.28 g L^–1^ day^–1^), compensating for the biomass decay during the night. This work provides guidelines to replicate this strategy and to apply it at higher scale. The control strategy here developed was proved to be effective on keeping bacteria contamination at negligible levels, equivalent to autotrophic cultures. The control of contamination allowed to attain organic substrate conversion to microalgae biomass with 0.54 biomass-to-substrate yield, comparable to axenic culture. The application of permeate from cheese whey ultrafiltration as fed gave less relevant improvements than pure glucose, likely due to the low efficiency of microalgae on using galactose. Using more effectively wastewater/byproducts as a source of organic substrate is the next step to make this process more environmentally and economically sustainable. To this aim, the employed wastewater should contain organic substrates that can be well metabolized by microalgae.

## Supplementary Material



## Notes

(Table S1) Modified M8 medium composition; (Table S2) HPLC elution program; (Table S3) permeate composition; (Figures S1, S2, S4, and S5) linear regressions; (Figure S3) optical density and biomass concentration correlations; (Figure S6) flow cytometry data (PDF)
