# Supplementary material for: Feast–Famine in Cyclic Autotrophy/Heterotrophy Doubles Microalgal Productivity while Controlling Bacterial Contamination
Source: ACS Sustain Chem Eng. 2025 Sep 26;13(39):16448–58. doi: 10.1021/acssuschemeng.5c05990 (PMC12506366; doi:10.1021/acssuschemeng.5c05990)
Supplement: Supplementary file 1 [file sc5c05990_si_001.pdf]

Supporting information

Feast-famine in cyclic autotrophy/heterotrophy doubles microalgal productivity while controlling bacterial contamination

Fabrizio Di Caprio*, Flavia Del Signore, Laura Capobianco, Francesca Pagnanelli, Pietro Altimari

Università Sapienza di Roma, Dipartimento di Chimica, Piazzale Aldo Moro 5, 00185, Rome, Italy.

*Corresponding Author: fabrizio.dicaprio@uniroma1.it

Number of pages: 6

Number of figures: 6

Number of tables: 3

Table of contents

Table S1. Composition of the modified M8 medium.

Table S2. HPLC elution program.

Table S3. Composition of permeate from ultrafiltration of whey.

Figure S1. Linear regression of growth data from photobioreactor vs time, excluding night phase.

Figure S2. Linear regression of growth data from photobioreactor vs time, including night phase.

Figure S3. Correlation between optical density and biomass concentration in different experiments.

Figure S4. Linear regression of growth data vs time in different experiments.

Figure S5. Linear regression of axenic bacteria and microalgae culture on different substrates.

Figure S6. Flow cytometry data of samples collected from different experiments.

Table S1: Chemical composition of the modified M8 medium used for the cultivation of *C. sorokiniana* in PBR.

Component	Modified M8
KH_2PO_4 (mM)	5.4
$Na_2HPO_4 \cdot 2H_2O$ (mM)	1.5
$MgSO_4 \cdot 7H_2O$ (mM)	1.6
$CaCl_2 \cdot 2H_2O$ (mM)	0.09
KNO_3 (mM)	29.7
EDTA ferric sodium salt (mM)	0.3
$Na_2EDTA \cdot 2H_2O$ (mM)	0.1
H_3BO_3 (mM)	0.001
$MnCl_2 \cdot 4H_2O$ (mM)	0.0656
$ZnSO_4 \cdot 7H_2O$ (mM)	0.0111
$CuSO_4 \cdot 5H_2O$ (mM)	0.0073

Table S2. Elution procedure used for the analysis of lutein.

Time	Acetonitrile (%)	methanol:ethyl acetate 1:1 (%)	200 mM acetic acid (%)
0	85	14.5	0.5
2	85	14.5	0.5
15	65	34.5	0.5
25	65	34.5	0.5

Table S3: Chemical composition of the permeate obtained from ultrafiltration of cheese whey (P_{UF}).

Component	P_{UF}
COD (g/L)	23.2 ± 0.2
Total sugars (g/L)	28 ± 8
Glucose (g/L)	9.6 ± 0.2
Total Nitrogen (mg/L)	180 ± 30
Total phosphorous (mg/L)	49.4 ± 0.3
pH	5.4 ± 0.4

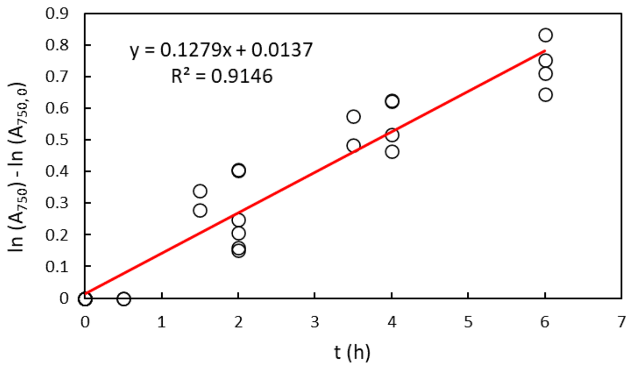

Figure S1. Linear regression of data of optical density collected in photobioreactors from two consecutive batches, plotted vs time. The time scale was rescaled to exclude night periods.

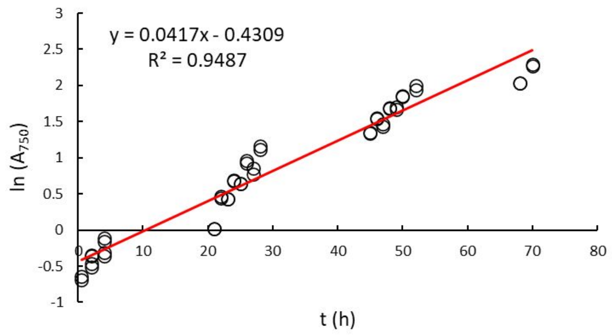

Figure S2. Linear regression of data of optical density collected in photobioreactors from two consecutive batches, plotted vs time. Night periods are included.

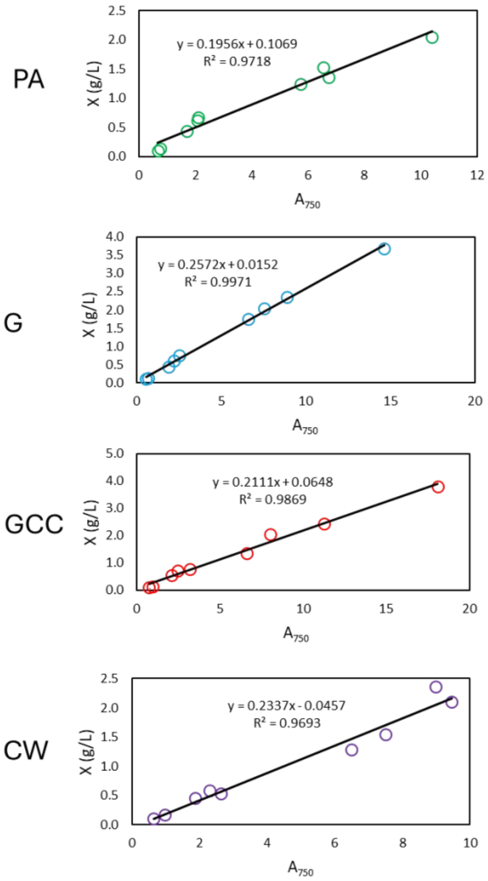

Figure S3. Correlation between A_{750} (optical density) and biomass dry weight (X) measured in the different experimental conditions tested.

Calculation of protein content (χ_{proteins}) by mass balance of total nitrogen consumption was made as follows:

$$\chi_{proteins}(\%) = \frac{C_{TN,0}V_0 - C_{TN,f}V_f}{X_f V_f - X_0 V_0} \, 100$$

With $C_{TN,0}$ initial nitrogen concentration, $C_{TN,f}$ final nitrogen concentration, V_0 and V_f initial volume and final volume, X_f and X_0 final biomass concentration and initial biomass concentration.

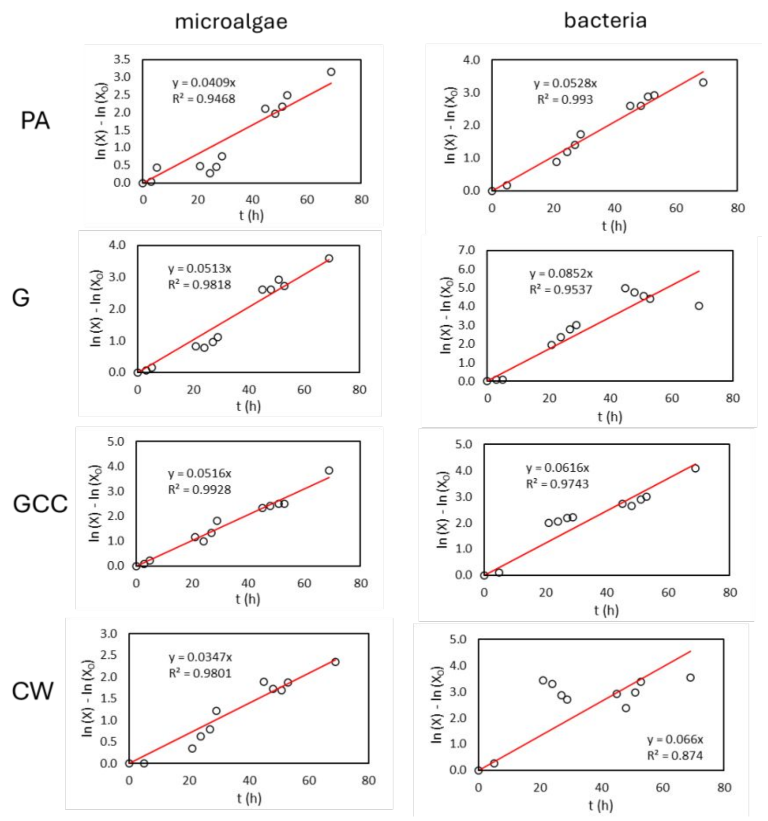

Figure S4. Linear regression of $\ln(X)$-$\ln(X_0)$ vs cultivation time for the different cultivation condition tested and for both microalgae and bacteria, using data of the whole cultivation, to calculate the average value of specific growth rate ($\mu_{M,avg}$, $\mu_{B,avg}$).

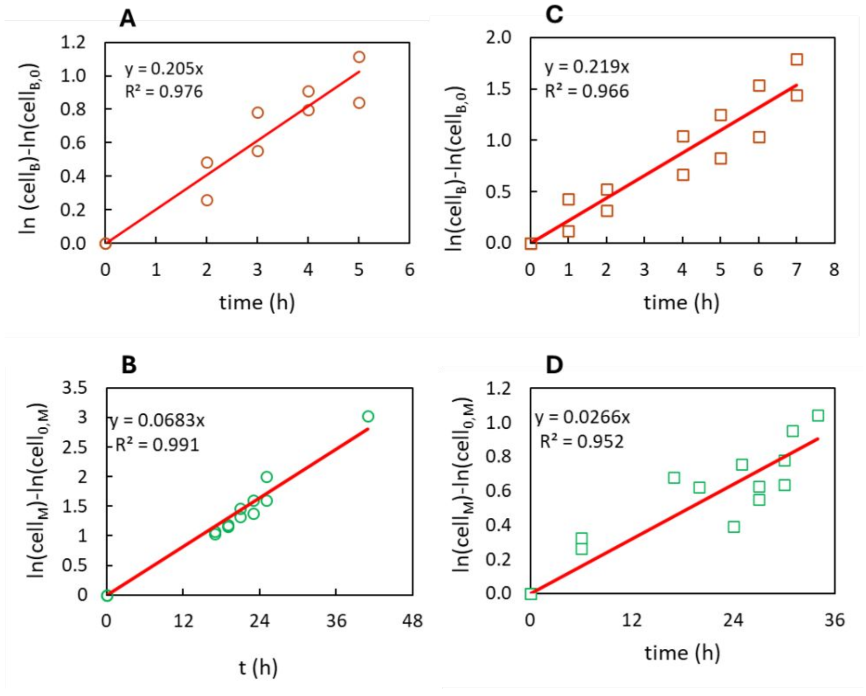

Figure S5. Linear regression of logarithmic cell concentration vs cultivation time for axenic microalgae *C. sorokiniana* (B and D) and bacteria flora living inside photobioreactors (A and B), on glucose (left, circles) and galactose (right, squares).

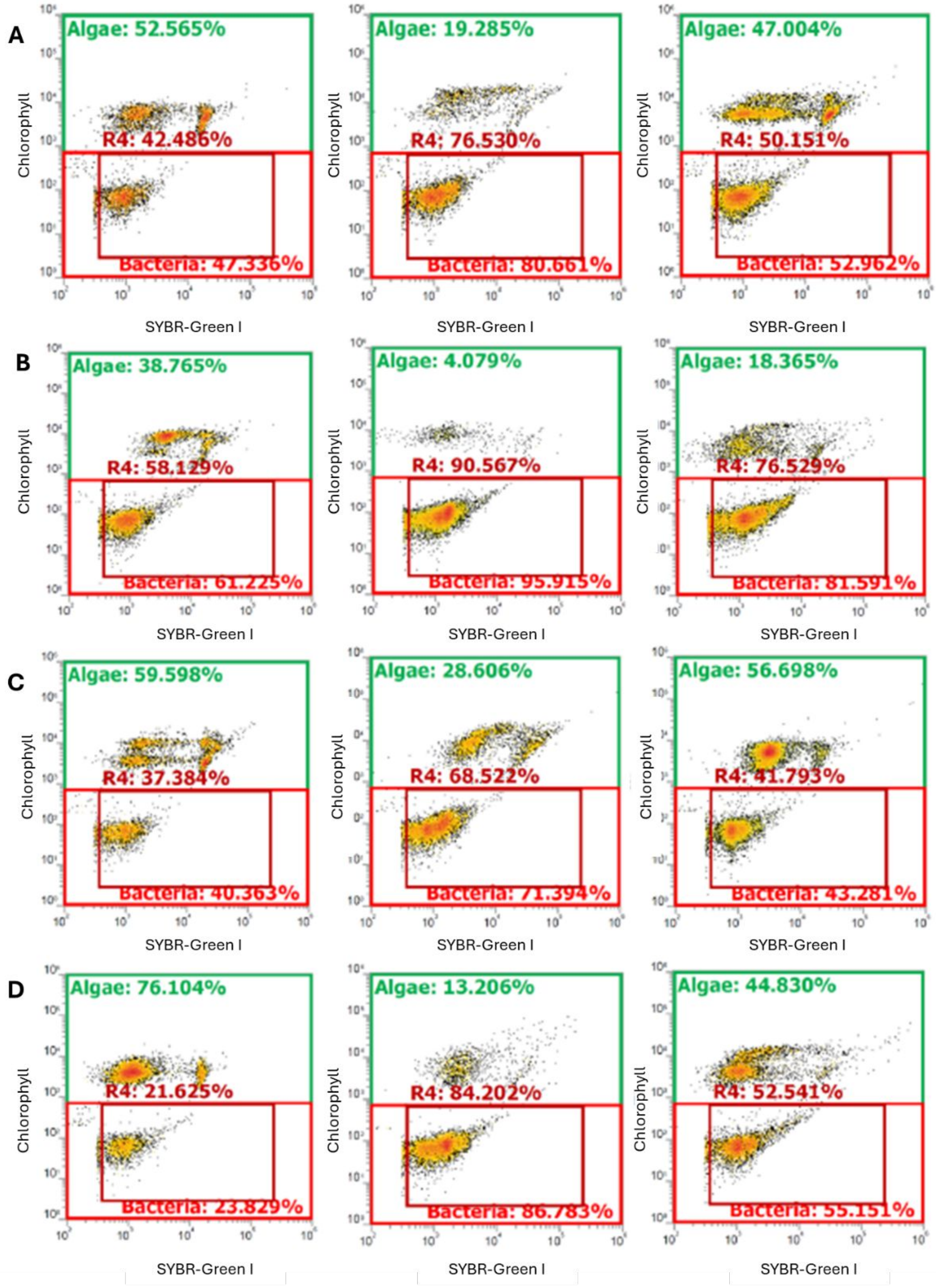

Figure S6. Scatter plot of data from flow cytometry analysis. Each line reports from left to right the data obtained for the starting point of the batch, the point at the maximum contamination and the final point of the batch, for the PA (A), G (B), GCC (C) and CWUP (D) condition.
